# The synergistic effect of *Ficus carica* nanoparticles and Praziquantel on mice infected by *Schistosoma mansoni* cercariae

**DOI:** 10.1038/s41598-024-68957-9

**Published:** 2024-08-15

**Authors:** Naira A. El-Attar, Mamdouh R. El-Sawi, Eman A. El-Shabasy

**Affiliations:** Zoology Department, Faculty of Science, Mansoura University, Mansoura City, Egypt

**Keywords:** C57BL/6 Mice, Protection, Fig, Nanoparticles, Schistosomiasis, Praziquantel, Gastrointestinal diseases, Infectious diseases, Nanobiotechnology, Biomarkers

## Abstract

Bilharzia is a parasitic flatworm that causes schistosomiasis, a neglected tropical illness worldwide. Praziquantel (PZQ) is a commercial single treatment of schistosomiasis so alternative drugs are needed to get rid of its side effects on the liver. The current study aimed to estimate the effective role of *Ficus carica* nanoparticles (Fc-NPCs), silver nanoparticles (Ag-NPCs) and *Ficus carica* nanoparticles loaded on silver nanoparticles (Fc-Ag NPCs) on C57BL/6 black female mice infected by *Schistosoma mansoni* and treated with PZQ treatment. It was proved that schistosomiasis causes liver damage in addition to the PZQ is ineffective as an anti-schistosomiasis; it is recorded in the infected mice group and PZQ treated group as in liver function tests, oxidative stress markers & anti-oxidants, pro-inflammatory markers, pro-apoptotic and anti-apoptotic markers also in liver cells’ DNA damage. The amelioration in all tested parameters has been clarified in nanoparticle-protected mice groups. The Fc-Ag NPCs + PZQ group recorded the best preemptive effects as anti-schistosomiasis. Fc-NPCs, Ag-NPCs and Fc-Ag NPCs could antagonize PZQ effects that were observed in amelioration of all tested parameters. The study showed the phytochemicals’ nanoparticles groups have an ameliorated effect on the health of infected mice.

## Introduction

Schistosomiasis is a parasitic disease caused by blood flukes of the genus *Schistosoma*. The disease has an acute phase that is followed by a chronic phase based on repeated exposure to the parasite if untreated. The main species causing human infections are *Schistosoma mansoni Schistosoma haematobium, Schistosoma japonicum* and *Schistosoma mekongi*. Approximately 779 million people are at risk of infection per year^1,2^.

Female C57BL/6 mice are usually used in severe diseases as hosts; female mice often show protection against most complement-driven injuries such as ischemia/reperfusion injury, graft rejection, bilharzia and sepsis, and this is caused by their strong immunity. Interestingly, early studies of the mouse complement system revealed that female mice have very low total complement activity (CH50) except this strain; this is related to androgen regulation of hepatic complement synthesis. and, they are not as nervous as males^3^.

Praziquantel (PZQ) is the only treatment against all *Schistosoma* species. The lack of a PZQ cure cannot prevent reinfection, causing fibrotic liver, and cannot affect eggs and larval stages highly encourage efforts to identify an alternative treatment for schistosomiasis, in anticipation of PZQ resistance and tolerance^4,5^. It is necessary to find natural alternatives to be efficient tools as new drugs against helminthiases, reducing the time and costs of drug research and development^6^. Its single advantage is killing 100% of only adult worms, now it has been recorded as 60–90%.

*Ficus carica* L. is known as one of the oldest trees and belongs to the Moraceae family, which is mostly cultivated in the Mediterranean territory. Its fruit is a rich source of sugar, vitamins, minerals, amino acids and organic acids^7^. Numerous bioactive compounds have been analyzed in leaves, including phenolic compounds, phytosterols, organic acids, the composition of anthocyanins, triterpenoids, coumarins, and volatile substances like hydrocarbons, aliphatic alcohols, as well as a few other classes of secondary metabolites^8^ which considered as antioxidant, anti-diabetic, anticancer, anti-inflammatory, hepatoprotective, anti-cancer, anti-cholinesterase, antimicrobial, antiviral activity and immune-enhancing properties^9^. Fig fruit, root, and leaves are used in traditional medicine to treat several conditions, including cardiovascular disorders, respiratory conditions (such as sore throats, coughs, and bronchial problems), and gastrointestinal conditions (such as colitis, indigestion, loss of appetite, and diarrhea)^10^. *Ficus carica* leaves active phytochemicals specifically phenolic compounds of such flavonoids (quercetin, rutin, luteolin) and phenolic acids (chlorogenic acid, caffeic acid); these compounds are known for their antioxidant properties which in turn stimulate immunity to fight the infection of schistosomiasis^11^.

Nanoparticles play a pivotal role since they exhibit unique properties; these properties depend on morphological aspects (e.g., shape, size, structure, crystallinity) and thus have led to a large range of applications in various areas such as electronics, molecular diagnostics, drug release and catalysis^12^. The biosynthesis strategy of nanoparticles has drawn much consideration instead of physical or chemical since it is green, proficiently manufactured forms, and cost-effective strategy. It will play a crucial role in diagnostics, drug delivery, bandages, related cosmetics, etc.^13^. The Ag-NPCs was used as antischistosomiasis as^14^ who compared silver nanoparticles (Ag NPs) with gold nanoparticles (Au NPs) in vitro and in vivo. The results observed that Ag NPs had the anti-miracidial, anti-cercarial and anti-schistosomal effects farther than Au NPs.

The present study aimed to evaluate the synergetic effect of Fc-NPCs, Ag-NPCs and Fc-Ag NPCs and PZQ treatment on C57BL/6 female mice infected by *S. mansoni* cercariae.

## Methods

### Ethical approval

The current study is reported by ARRIVE guidelines. The experimental protocol was agreed upon by the local experimental animal ethics committee of the Faculty of Medicine, Mansoura University accordance with the guide of the National Institute of Health for the care and use of Laboratory animals (NIH publication No. 8523, revised 1996) with approval number Sc. Ms. 22.12.12. The date of approval: is 16/12/2022.

### Chemicals

Praziquantel (PZQ) tablets were purchased from the Egyptian International Pharmaceutical Industries Company (EPICIO), Mansoura, Egypt.

### Mice groups and mode of administration

**Figure 1:**
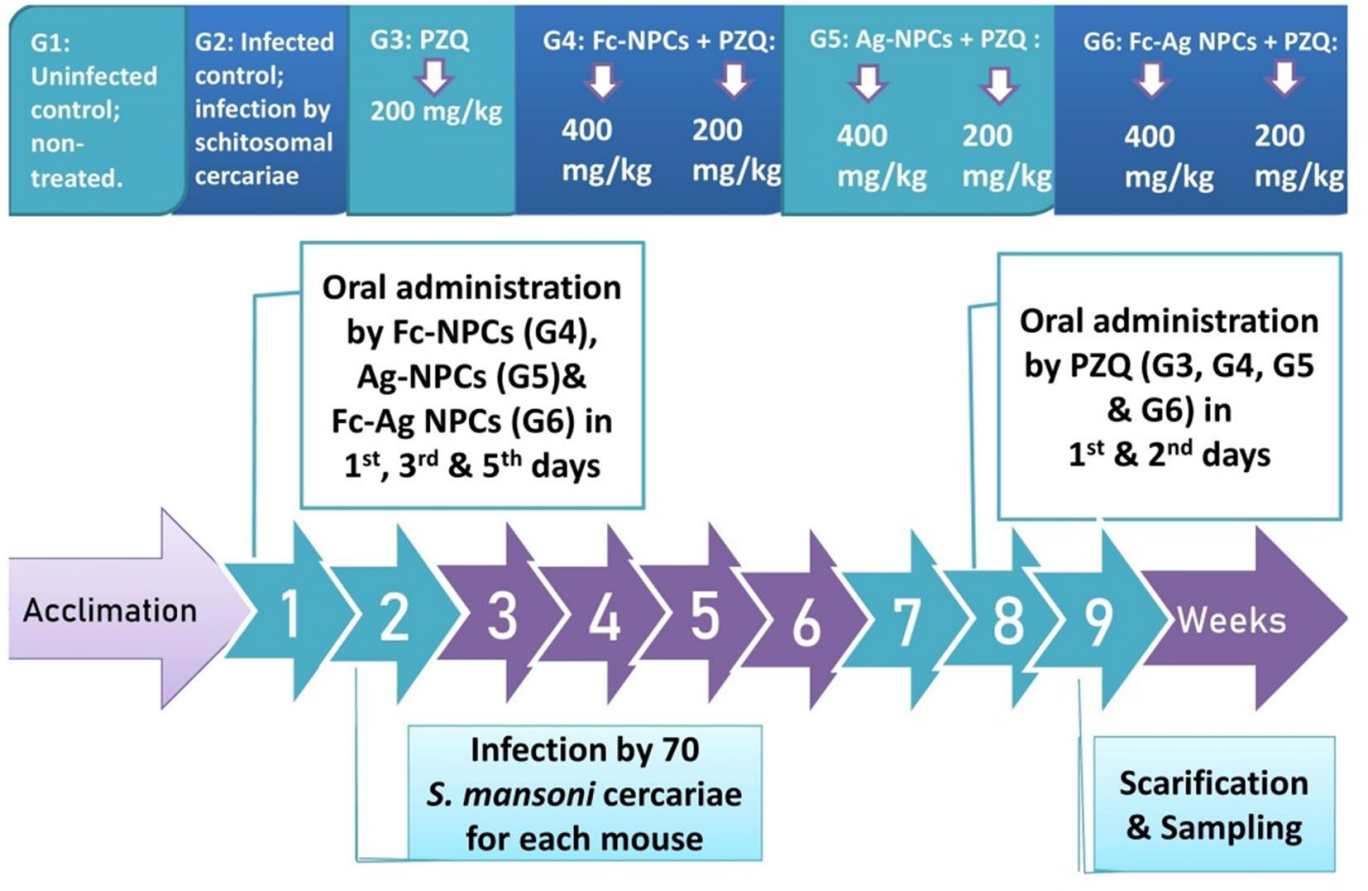
Experimental design for animal groups; PZQ: Praziquantel, Fc-NPCs: *Ficus carica* nanoparticles, Ag-NPCs: Silver nanoparticles and Fc-Ag NPCs: *Ficus carica* nanoparticles loaded on silver nanoparticles.

Thirty female mice C57BL/6 aged 11 weeks with an average weight of 32.5 g were cared for wood-chip bedding plastic cages, refreshed day by day for acclimation with standard food as pellets of commercial rodents’ food and water ad libitum at experimental period, at 22 ± 3 ℃ for 12 h cycle dark/ 12 h light in animal house of the Faculty of Science, Mansoura University, Mansoura, Egypt. Mice were randomly divided into six groups (n = 5) during the experimental period = 8 weeks which was summarized in Fig. 1:

- **G1-**Uninfected control group: Uninfected and non-treated.
- **G2-**Infected control group: Infected by *S. mansoni* cercariae- non-treated.
- **G3-** PZQ group: 200 mg/Kg for two consecutive days was given orally by oral tube during the 7th week after *S. mansoni* cercariae infection^14^.
- **G4-** Fc-NPCs + PZQ: Mice were pre-treated by Fc-NPCs (400 mg/Kg) through oral gavage in 1st, the 3rd and 5th days during 1st week before *S. mansoni* cercariae infection, PZQ was administrated orally for two consecutive days (200 mg/day) during 7th week after infection^15^.
- **G5-** Ag-NPCs + PZQ: Mice were pre-treated by Ag-NPCs (400 mg/Kg) through oral gavage in 1st, the 3rd and 5th days during 1st week before *S. mansoni* cercariae infection, PZQ was administrated for two consecutive days (200 mg/day) orally during 7th week after infection^15^.
- **G6-** Fc-Ag NPCs + PZQ: Mice were pre-treated by Fc-Ag NPCs (400 mg/Kg) through oral gavage in 1st, the 3rd and 5th days during 1st week before *S. mansoni* cercariae infection, PZQ was administrated for two consecutive days (200 mg/day) orally during 7th week after infection^15^.

All infected mice were injected subcutaneously by freshly shedding *S. mansoni* cercariae from a stock solution containing about 70 cercariae/ 0.5 ml distilled water.

### Preparation of praziquantel stock solution (Fresh)

Two hundred mg of PZQ was mixed with a few drops of highly purified olive oil until reaching a paste texture, and then 5.2 ml of sterile water was added drop by drop with continuous stirring.

### *Ficus carica* extract nanoparticles preparation

Hydrothermal squeeze methods were confirmed for the preparation process; 20 g of washed and dried powdered leaves plant were soaked in 100 ml ethanol for 12 h. in the absence of light. In a Teflon-lined autoclave, samples were been introduced and placed in an incubator at 50 ± 2 ℃ for 6 h. After the incubator, the solutions were kept cool to room temperature for another 6 h. Then filtered, centrifuged, and freeze-dried. The process is repeated until the required amount of extract is obtained^16^.

### Preparation of silver nanoparticles

About 10 ml of prepared *F. carica* extract was added to 100 ml of 2 mM of a pre-prepared aqueous solution of silver nitrate according to the Green synthesis technique. The solution was stirred at 60 ℃ for 6 h. The formation of the Ag-NPCs was detected by the appearance of deep brown^17^.

### Preparation of Fig extract nanoparticles loaded on silver nanoparticles

By adding 1:1 ethanoic *F. carica* extract: Silver nitrate, with continuous stirring for 1 h. at pH = 7, after 24 h. in the dark, with stirring and then left until yellowish-green color appears.

### Characterization of nanoparticles

The Fc-NPCs, Ag-NPC and Fc-Ag NPCs were characterized by measuring Zeta-potential and hydrodynamic size (DLS) using the Malvern Zetasizer instrument, Malvern Panalytical Company, Worcestershire, United Kingdom.

### Scarification, blood sampling and hepatic samples preparation

At the end of the experiment, mice were drugged by Ketamine (43.5 mg/ kg) and Xylazine (6.5 mg/ kg) for half hr. to be fully euthanized^18^. Mice were sacrificed by decapitation using sharp, cleaned and sterilized blades (The decapitation was used for collecting blood samples to be followed by dissection to obtain the liver for other investigations) and blood samples were obtained in centrifuge glass tubes, after clotting, samples were centrifuged at 3000 r.p.m. for quarter of hr., the clear supernatant was collected. The sera were kept in labeled Eppendorf’s tubes, frozen at −20 ℃ until usage. Hepatic samples were washed, dried and homogenized (10% w/v) with distilled water; homogenates were stored in labeled Eppendorf tubes at −20 ℃ for biochemical analyses.

## Physiological markers

### Liver function tests

Serum alanine aminotransferase (ALT), aspartate aminotransferase (AST) and alkaline phosphatase (ALP) activities have been evaluated according to the colorimetric kit methods of^19,20^, respectively using a Biodiagnostic and Research reagents kit. Serum gamma-glutamyl transferase (GGT) activity has been evaluated according to the colorimetric kit method of^21^, using a kit from Clinical Chemistry. Serum albumin and bilirubin contents were investigated according to the methods described by^22,23^, respectively using of kit from Spinreact.

### Oxidative stress and antioxidant markers

Hepatic malondialdehyde (MDA), reduced glutathione (GSH), superoxide dismutase (SOD) and catalase (CAT) were investigated according to the methods described by^24–27^, respectively by the use of kit from Biodiagnostic and Research reagents.

### Pro-inflammatory markers

Serum C reactive protein (CRP) was estimated using an ELISA kit from BD™ ELISA, Franklin Lakes, New Jersey, USA. Hepatic interleukin-6 (IL-6) and vascular adhesion molecule-1 (VCAM-1) were estimated by ELISA kit from CUSABIO, Houston, TX, USA. Intercellular adhesion molecule-1 (ICAM-1) was investigated using an ELISA kit from Quantikine®ELISA, Riham Tower El-Etehad Square, Maadi, Cairo, Egypt.

### Pro-apoptotic and anti-apoptotic markers

Hepatic p53, Bax, cytochrome c and caspase 9 were estimated by ELISA kit from Rat P53/Tumor Protein (P53/TP53) ELISA Kit, ELISA kit from Rat apoptosis regulator BAX (BAX) ELISA Kit, ELISA kit and ELISA kit from Rat CASP9 (Caspase 9) ELISA Kit, respectively CUBIO Innovation Center, Houston, TX, USA. Hepatic Bcl-2 was estimated by ELISA kit from Rat B-cell CLL/lymphoma 2 (BCL2) ELISA Kit, Creative Bio labs, Ramsey Road, Shirley, USA. Hepatic caspase-3 was estimated by using an ELISA kit from Biovision, Grove Street, Waltham, Massachusetts.

### DNA damage by single cell electrophoresis assay

The DNA damage was investigated by using hepatic cells according to^28,29^.

### Statistical analyses

The GraphPad Prism 8.0 software, version number 8.0 (Graphpad Software Inc., San Diego, California, USA) was used for preparing all statistics. The results are shown as the mean ± standard error of the mean (SEM) (n = 5). A one-way analysis of variance (ANOVA) was used to make the different statistical comparisons, followed by the Neuman-Keuls post-hoc test. The data was considered significant when *P* ≤ 0.05^30^. The URL link of GraphPad: https://www.graphpad.com/myaccount/subscription/?subscriptionID=2878432

## Results and discussion

### Characterization of nanoparticles of samples

#### Zeta potential and size of *Ficus carica* nanoparticles

**Figure 2:**
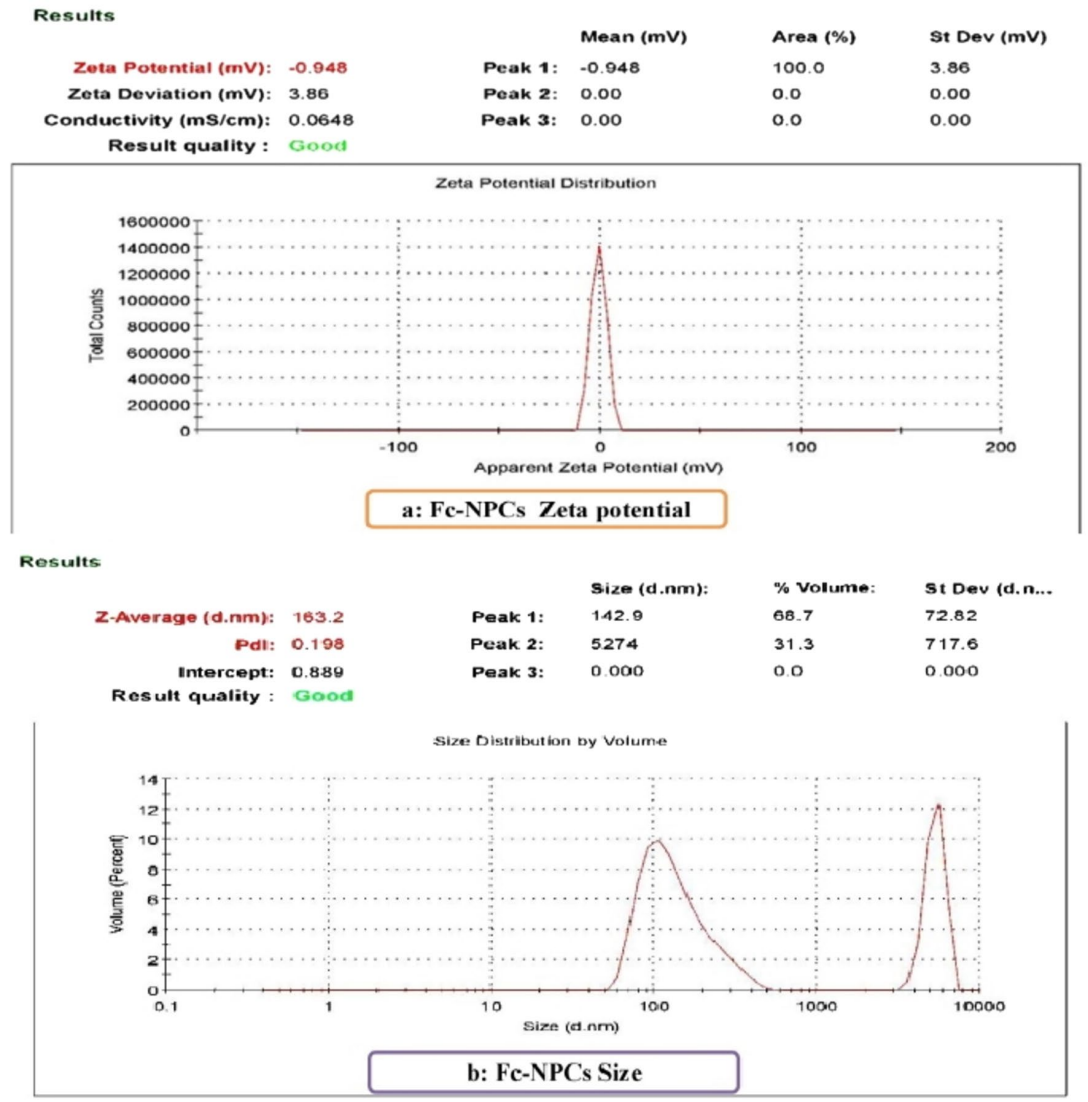
The characterization of Fc-NPCs as; (**a**): Zeta-potential and (**b**): Size of nanoparticles.

The charge of nanoparticles of *Ficus carica* leaves extract with a mean -0.948 mV was shown in graph a (Fig. 2). While its size average was 163.2 nm as in graph b (Fig. 2).

#### Zeta potential and size of silver nanoparticles

**Figure 3:**
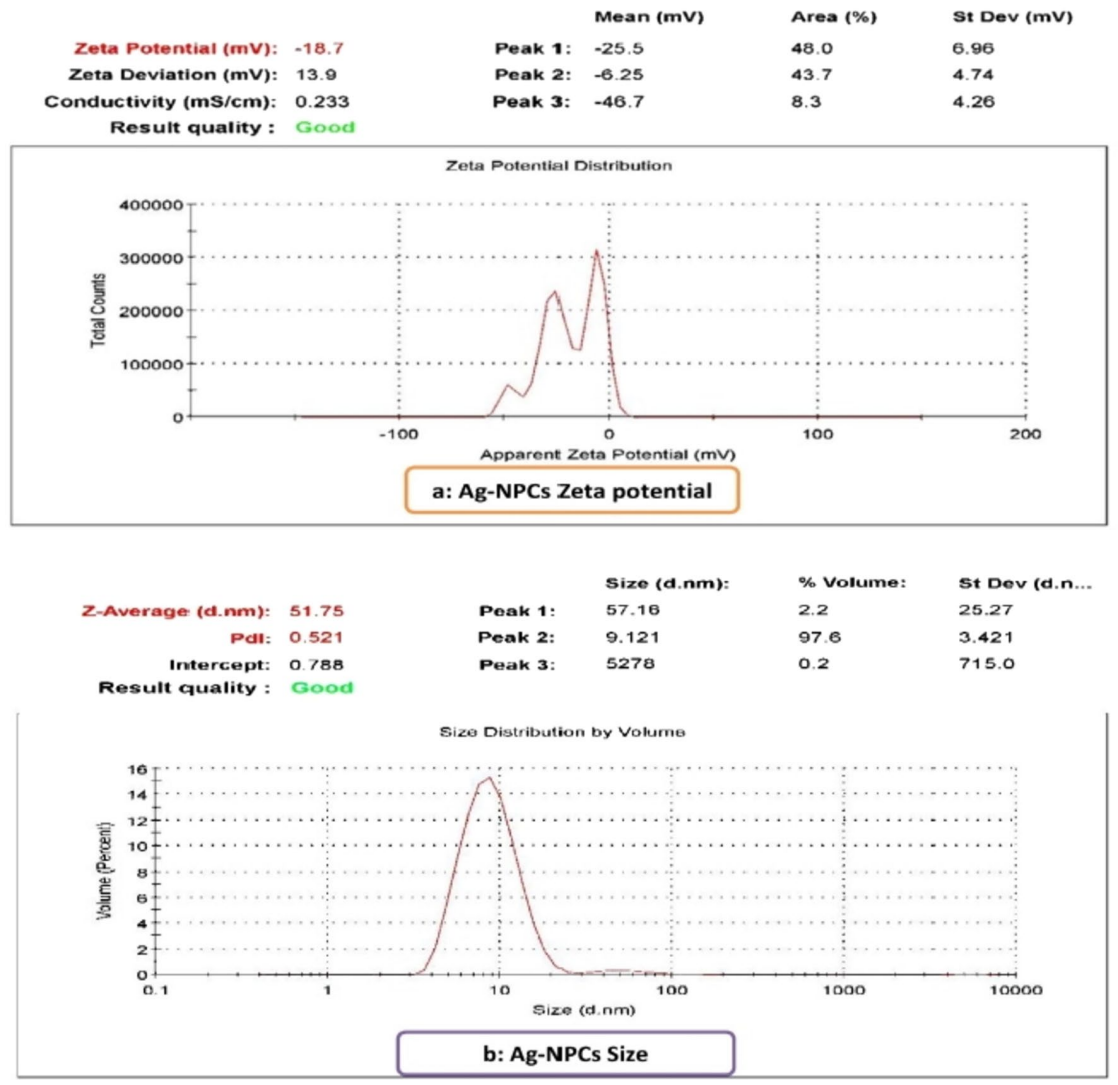
Characterization of Ag-NPCs as **(a)**: Zeta potential and **(b)**: Size of nanoparticles.

The charge of nanoparticles of silver with a mean −18.7 mV was shown in graph a (Fig. 3). While its size average was 51.75 nm as in graph b (Fig. 3).

#### Zeta potential and size of *Ficus carica* nanoparticles loaded on silver nanoparticles

**Figure 4:**
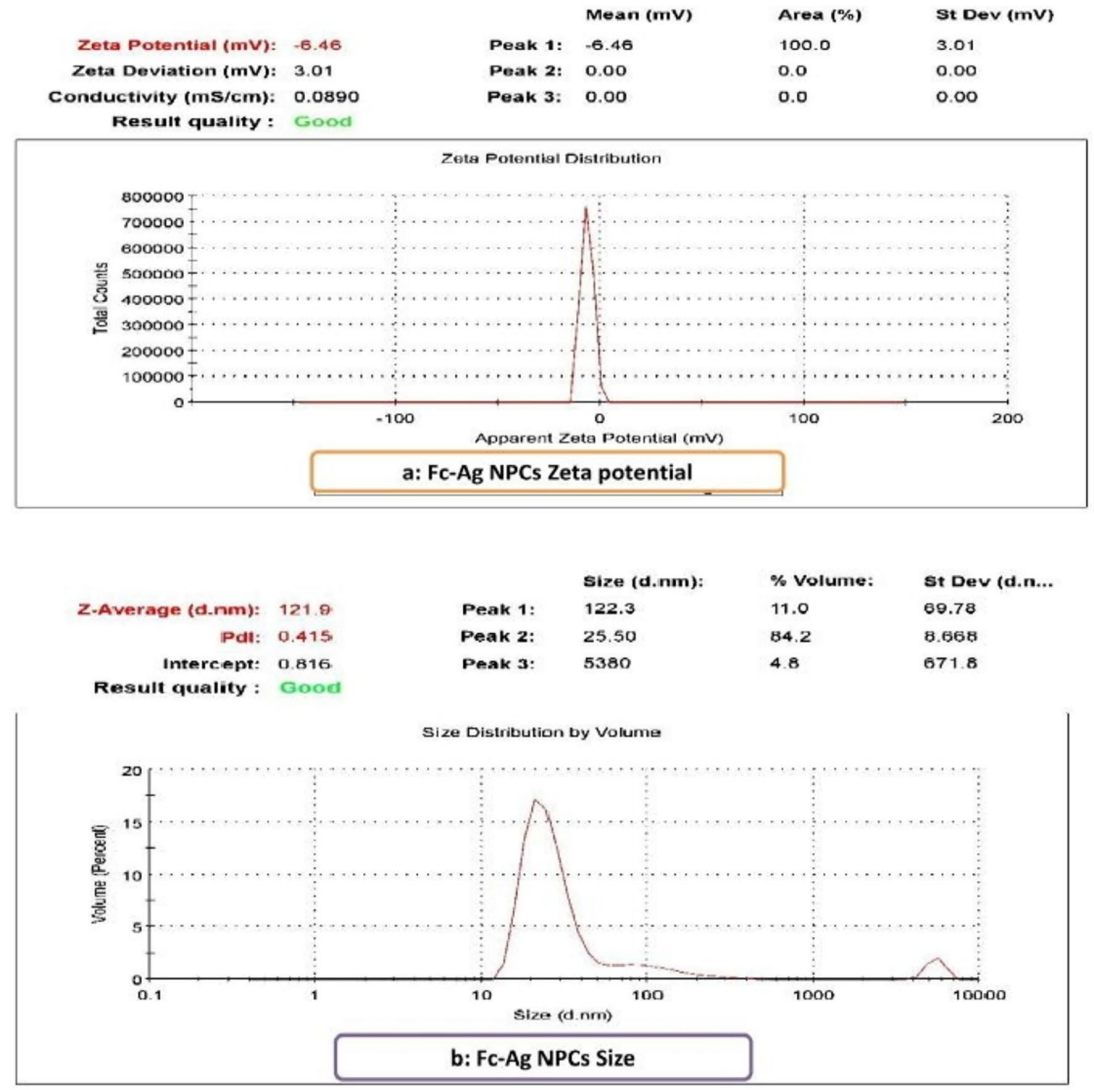
Characterization of Fc-Ag NPCs as (**a)**: Zeta potential and (**b)**: Size of nanoparticles.

The charge of nanoparticles of *Ficus carica* leaves extract loaded on silver nanoparticles with −6.46 mV was shown in graph a (Fig. 4). While its size average was 121.9 nm as in graph b (Fig. 4).

### Liver function tests

**Figure 5:**
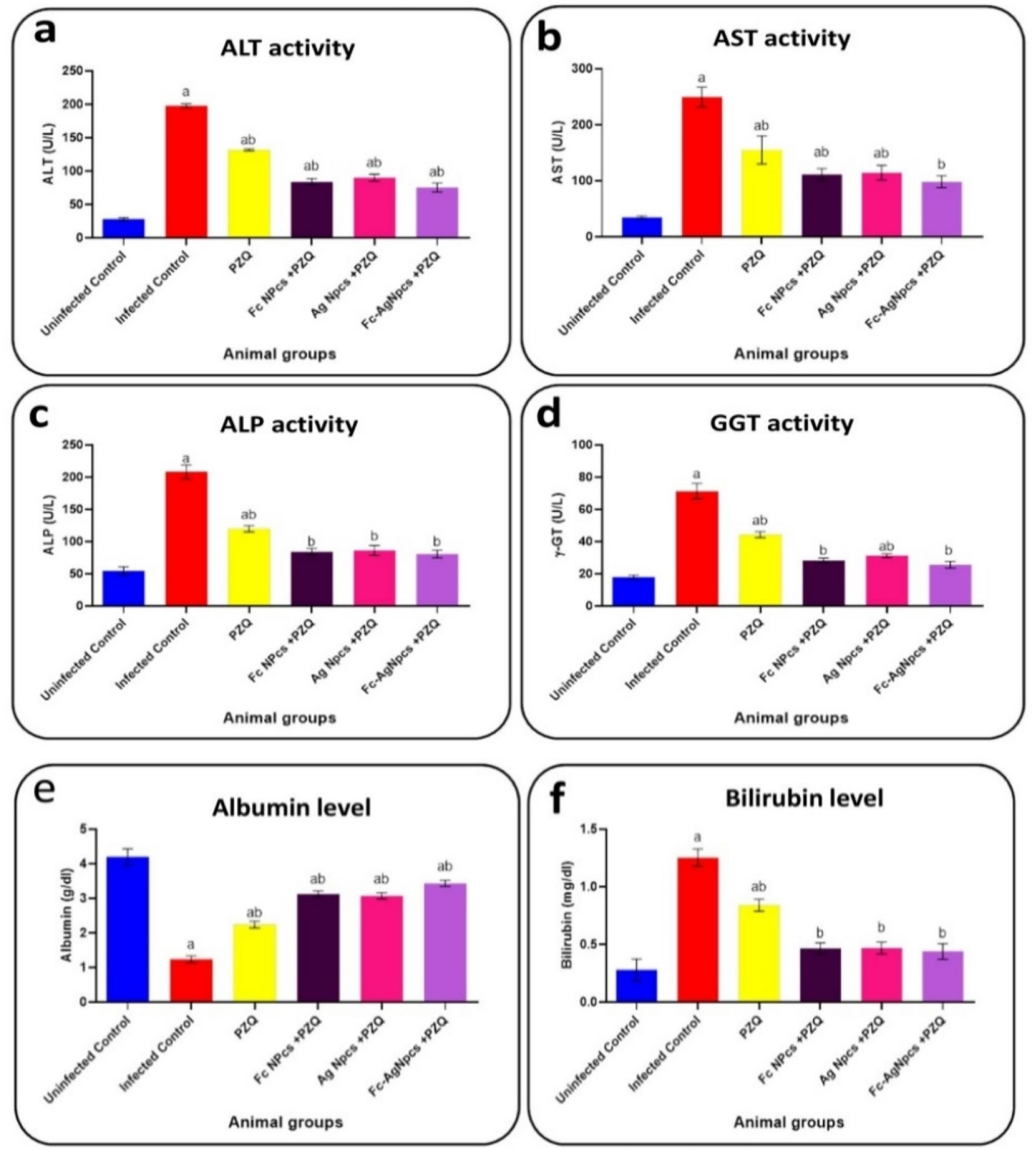
Liver function tests as; (**a**): Alanine aminotransferase, (**b**): Aspartate aminotransferase, (**c**): Alkaline phosphatase, (**d**): Gamma-glutamyl transferase, (**e**): Albumin & (**f**): Bilirubin in PZQ (Praziquantel), Fc-NPCs (*Ficus carica* nanoparticles), Ag-NPCs (Silver nanoparticles) & Fc-Ag NPCs (*Ficus carica* nanoparticles loaded on silver nanoparticles).

Figure 5a–f observed the activity of each ALT, AST, ALP, GGT, content of each albumin and bilirubin in control and tested animal groups.

#### The effect of different nanoparticle groups on liver function tests

Serum activity of ALT, AST, ALP, GGT and content of bilirubin recorded significant reduction unlike albumin content recorded a significant increase in Ag-NPCs, Fc-NPCs and Fc-Ag NPCs pre-treated groups compared to infected mice.

#### Praziquantel treatment effects on liver function tests

PZQ showed a significant effect on the above serum markers compared with the + ve control group that had an agreement with^31^ who explained the increment of such enzymes in serum is probably due to the destruction of hepatocytes by the action of parasite eggs-releasing toxins with low effectiveness of PZQ drug in full recovery additionally enhancing fibrotic liver.

#### The effect of *Ficus carica* nanoparticles on liver function tests

The dose of 400 mg/kg of *Ficus carica* ethanolic leaves extract nanoparticles did not record any death cases as in the^11^ study that used the same dose of ethanolic extract. These results had an agreement with^32^ who stated that it was approved that using PZQ (sub-curative dose) in combination with other anti-schistosomicides such as *F. carica* leaves ethanolic extract is an important step to reduce the PZQ dose used and avoid side effects.

#### The effect of silver nanoparticles on liver function tests

In the current study, there were no deaths have been recorded by using the dose of 400 mg/kg by Ag-NPCs which was a safe dose^33^ noted that Ag-NPCs could reduce liver AST, ALT, and ALP. From the obtained findings, it may be due to the green synthesis method which reduced the toxicity of silver to be safer, indicating a potential protective role of Ag-NPCs.

#### The effect of *Ficus carica* nanoparticles loaded on silver nanoparticles on liver function tests

The group that showed the best-recovered liver after cercarial infection is Fc-Ag NPCs + PZQ; it may come from Fc-NPCs and Ag-NPCs synergizing each other and reducing the side effects of a sub-curative dose of PZQ treatment.

### Oxidative stress marker & Anti-oxidants

**Figure 6:**
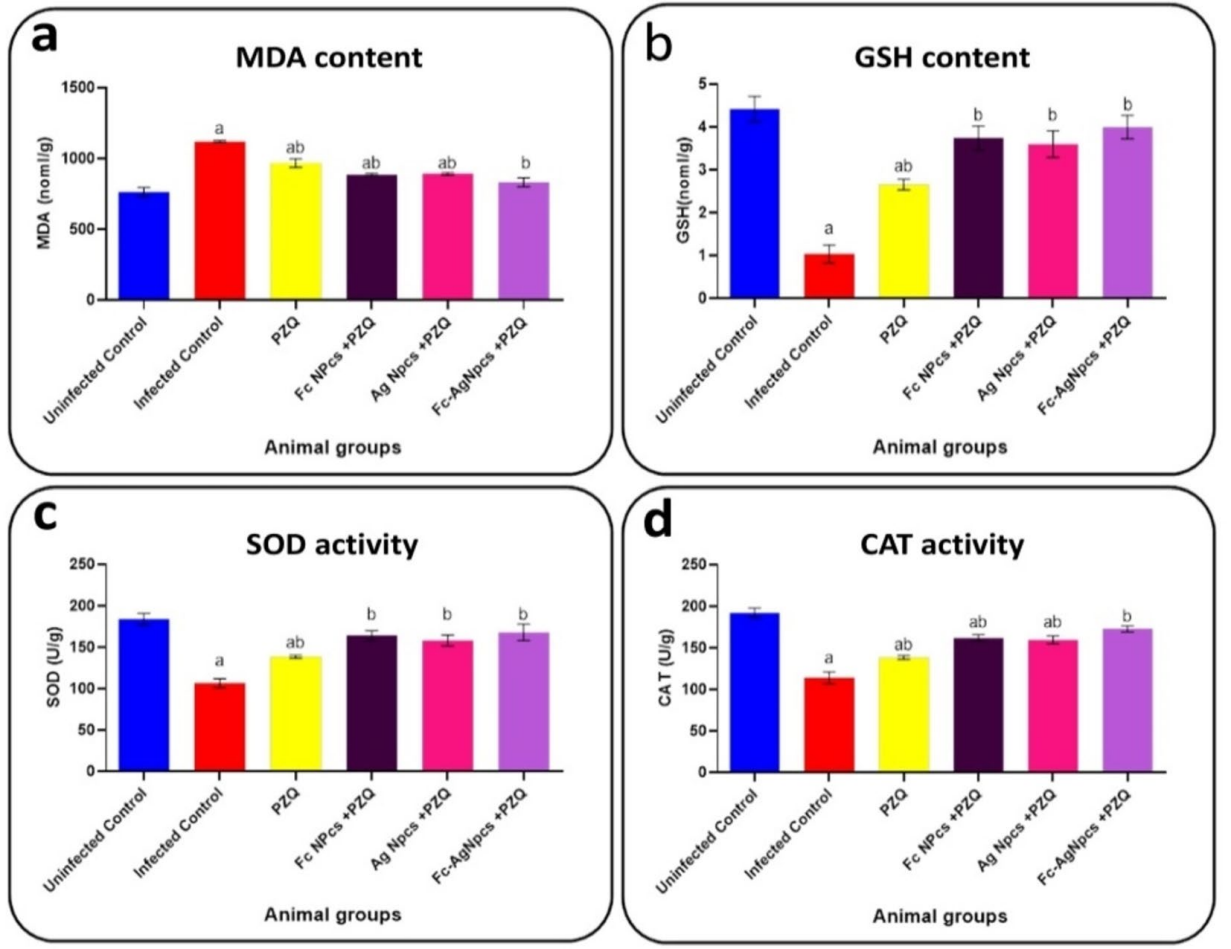
Oxidative stress marker & Anti-oxidants as; (**a**): Malondialdehyde, (**b**): Reduced glutathione, (**c**): Superoxide dismutase & (**d**): Catalase in PZQ (Praziquantel), Fc-NPCs (*Ficus carica* nanoparticles), Ag-NPCs (Silver nanoparticles) & Fc-Ag NPCs (*Ficus carica* nanoparticles loaded on silver nanoparticles).

Figure 6a–d observed content of each MDA and GSH, the activity of each SOD and CAT in control and tested animal groups.

#### The effect of different nanoparticle groups on oxidative stress and anti-oxidant markers

Hepatic level of MDA showed a significant decrease contrary to GSH level and activity of SOD and CAT recorded significant elevation in PZQ, Ag-NPCs, Fc-NPCs and Fc-Ag NPCs pre-treated groups compared to the infected group.

#### Praziquantel treatment effects on oxidative stress marker & anti-oxidants

Praziquantel showed low effectiveness in full recovery and appeared in its lower amelioration on liver function tests and also in anti-oxidants contrary to oxidative stress markers.

#### The effect of *Ficus carica* nanoparticles on oxidative stress marker & anti-oxidants

Natural antioxidant compounds of *Ficus carica* leaves such as phenolic compounds, organic acids, vitamin E, and carotenoids are healthy compounds; these compounds can inhibit free radical formation by reducing or donating hydrogen to other compounds. Among them, phenolic compounds are the most popular due to their well-known antioxidant capacities. Two major categories of phenolic compounds are phenolic acids and flavonoids^34^.

#### The effect of silver nanoparticles on oxidative stress marker & anti-oxidants

Reference^35^ had the same conclusion and explained that it may restore the GSH found in the liver in Ag-NPs treated animals. Alleviated activities of adenosine triphosphatase (ATPase), glucose-6- phosphatase (G6Pase) and antioxidant enzymes viz. SOD and CAT due to acetaminophen (APAP) induced toxicity in the liver were recovered by the treatment of Ag-NPs.

#### The effect of *Ficus carica* nanoparticles loaded on silver nanoparticles on oxidative stress marker & anti-oxidants

Reference^34,36^ who stated the ameliorative effect of fig nanoparticles and silver nanoparticles in addition to their effect on PZQ treatment to antagonize it so, this combined group marked the best effect on the anti-oxidation process.

### Pro-inflammatory markers

**Figure 7:**
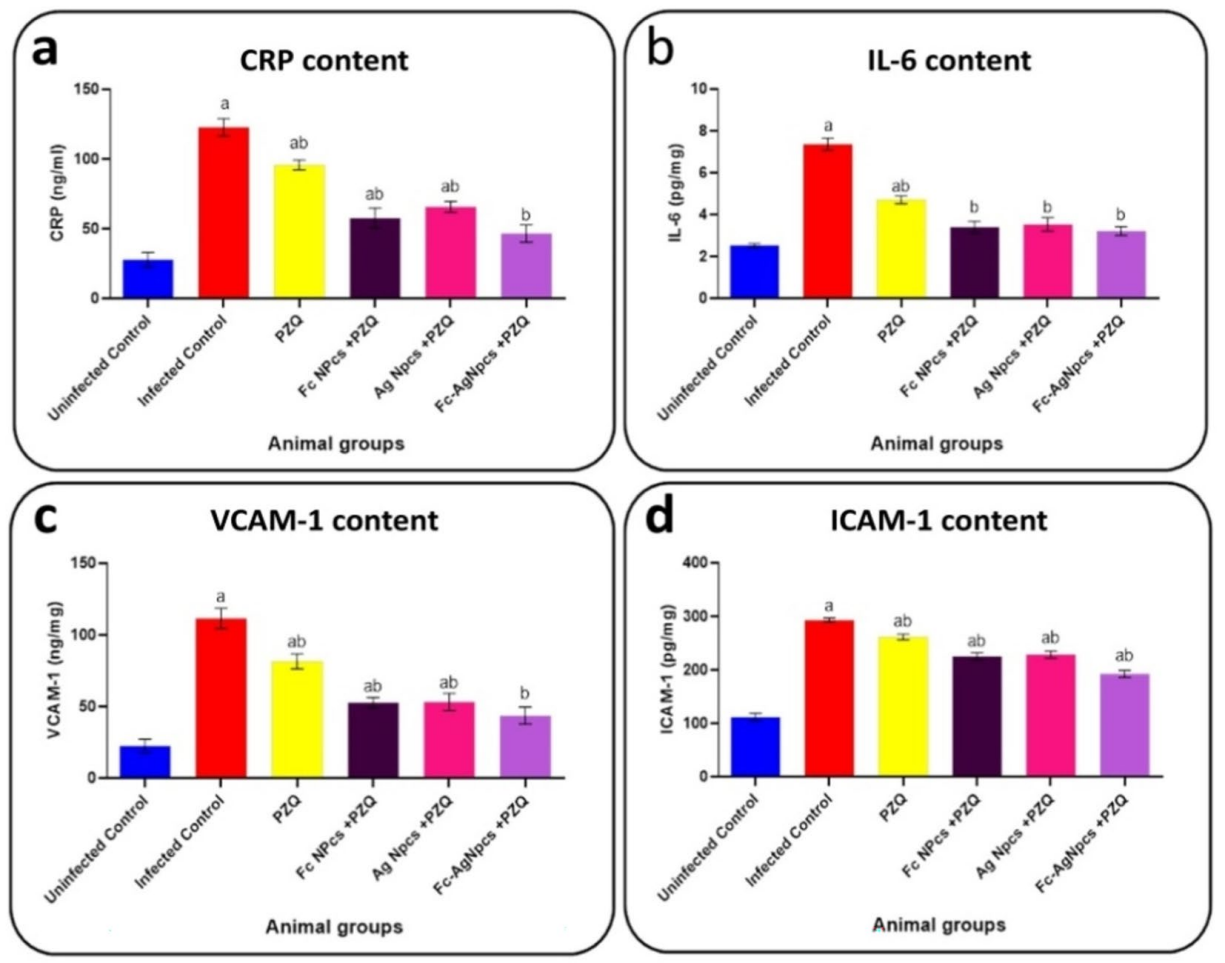
Pro-inflammatory as; (**a**): C-reactive protein, (**b**): Interleukin-6, (**c**): Vascular adhesion molecule-1 & (**d**): Intercellular adhesion molecule-1 in PZQ (Praziquantel), Fc-NPCs (*Ficus carica* nanoparticles), Ag-NPCs (Silver nanoparticles) & Fc-Ag NPCs (*Ficus carica* nanoparticles loaded on silver nanoparticles).

Figure 7a–d observed the content of each CRP, IL-6, VCAM-1 and ICAM-1 in control and tested animal groups.

#### The effect of different nanoparticle groups on inflammatory markers

Serum CRP, hepatic IL-6, VCAM-1 and ICAM-1 showed significant decrease in PZQ, Ag-NPCs, Fc-NPCs and Fc-Ag NPCs pre-treated mice groups compared with the infected group.

#### Praziquantel treatment effects on inflammatory markers

Praziquantel appeared to have limitations in achieving full recovery, improving liver function tests, and balancing antioxidant levels with oxidative stress markers. Its effect on pro-inflammatory markers suggested a specific impact on inflammation, which could be fatal depending on the condition.

#### The effect of *Ficus carica* nanoparticles on inflammatory markers

Oxidative stress was closely related to inflammatory response since unbalance of redox homeostasis can cause cellular damage, thus activating the innate immune response or causing apoptosis^37^. Formononetin (one of the phytochemicals of *F. carica*) indicated that it had a positive effect, as it increased the activity of antioxidant enzymes, and reduced the levels of pro-inflammatory markers (TNF-α, IL-1β, and IL-6) via HMGB1/TLR4/NF-κB signaling and NLRP3 inflammasome pathways^38^.

#### The effect of silver nanoparticles on inflammatory markers

Reference^39^ explained their results by using Ag-NPCs to ameliorate liver inflammation and damage by reducing macrophage and neutrophil infiltrates. A reduction in count and size was evaluated in the granulomas, as well as a change to an exudative-proliferative phase, with a local increase of IFN-γ (anti-tumor).

#### The effect of *Ficus carica* nanoparticles loaded on silver nanoparticles on inflammatory markers

From the previous explanations about the ameliorative effects of Fc-NPCs, Ag-NPCs and PZQ on liver function tests, as antioxidants, and anti-inflammatory agents, and the recorded data observed the best anti-inflammatory treatment in this group that may be caused by the nanoparticles can synergize each other and antagonize PZQ effects.

### Pro-apoptotic and anti-apoptotic markers

**Figure 8:**
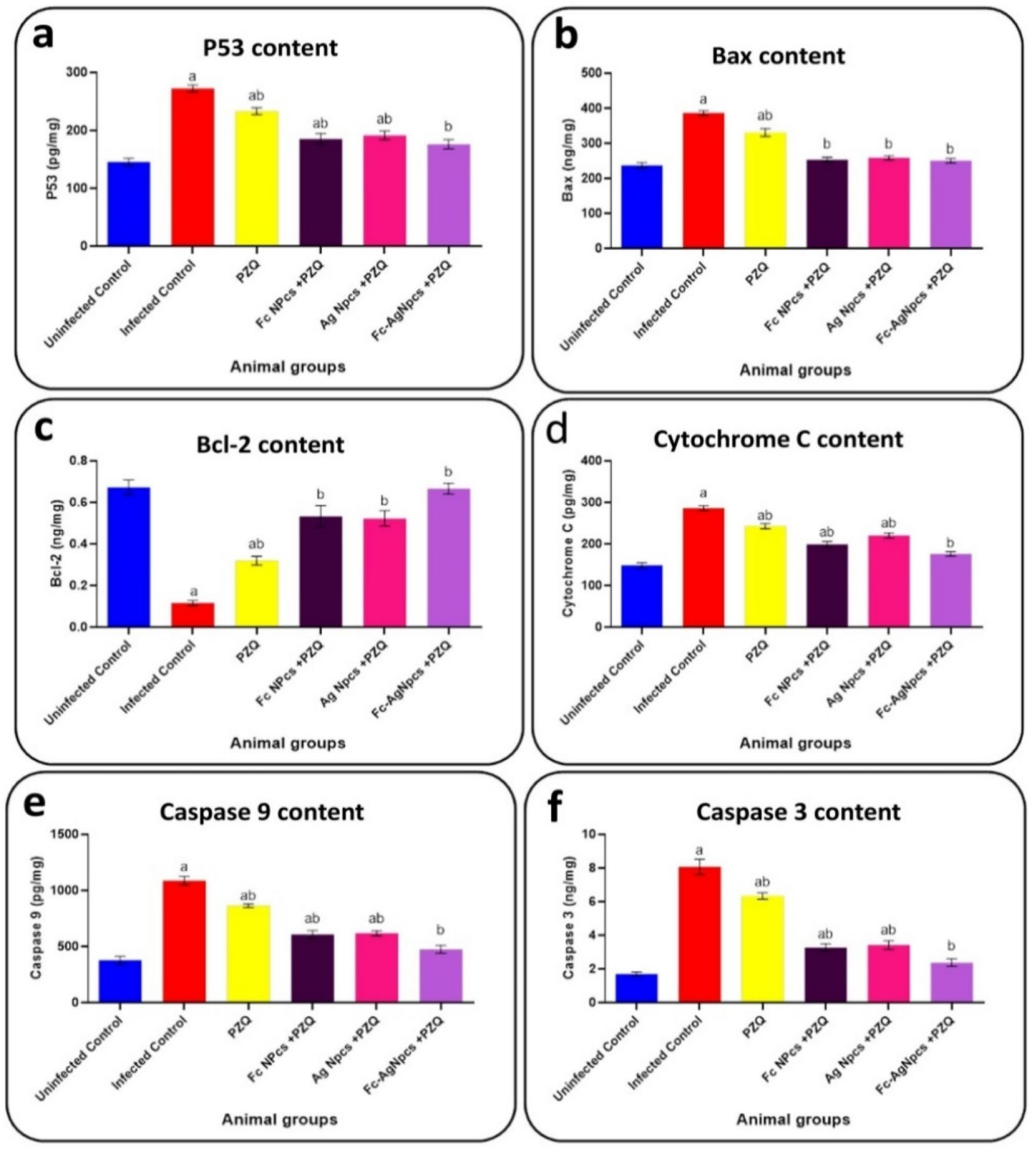
Pro-apoptotic and anti-apoptotic markers as; (**a**): P53, (**b**): Bax, (**c**): Bcl-2, (**d**): Cytochrome C, (**e**): Caspase 9 & (**f**): Caspase 3 in PZQ (Praziquantel), Fc-NPCs (*Ficus carica* nanoparticles), Ag-NPCs (Silver nanoparticles) & Fc-Ag NPCs (*Ficus carica* nanoparticles loaded on silver nanoparticles).

Figure 8a–f observed the content of each P53, Bax, Bcl-2, cytochrome C, caspase 9 and caspase 3 in control and tested animal groups.

#### The effect of different nanoparticle groups on apoptotic and anti-apoptotic markers

Hepatic P53, Bax, cytochrome C, caspase 9 and caspase 3 showed significant decrease otherwise Bcl-2 recorded a significant increase in PZQ, Ag-NPCs, Fc-NPCs and Fc-Ag NPCs pre-treated mice groups compared with the infected group.

#### Praziquantel treatment effects on pro-apoptotic and anti-apoptotic markers

Praziquantel appeared to have complex effects that include limitations in achieving full recovery, improving liver function tests, and balancing antioxidant levels with oxidative stress markers. Its impact on pro-inflammatory and apoptotic markers suggested dangerous influences, which could be mortal by *S. mansoni* infection based on condition.

#### The effect of *Ficus carica* nanoparticles on pro-apoptotic and anti-apoptotic markers

Reference^40^ who used *Ziziphus spina-christi* leaf extract (ZLE) that had polyphenols as Fc-NPCs; the results indicated that ZLE had anti-fibrotic activity by reversing *S. mansoni*-induced liver fibrosis through reducing hepatic expression of TGF-β1, MMP-9, and TIMP-1 and increasing anti-oxidative status while limiting apoptosis. ZLE had anti-schistosomal activity, in addition to antioxidant, anti-inflammatory, anti-apoptotic and anti-fibrotic effects.

#### The effect of silver nanoparticles on pro-apoptotic and anti-apoptotic markers

Reference^41^ who explained that Ag-NPCs had antioxidant, anti-inflammatory, and anti-apoptotic effects as mentioned above. The presence of Ag-NPCs might improve the effect of PZQ so the obtained result showed a better effect than PZQ singly.

#### The effect of *Ficus carica* nanoparticles loaded on silver nanoparticles on pro-apoptotic and anti-apoptotic markers

From the previous data about the ameliorative effects of Fc-NPCs and Ag-NPCs as antioxidants, anti-inflammatory and anti-apoptotic agents, and the recorded data observed the best anti-apoptotic treatment in this group (Fc-Ag NPCs + PZQ) that may be caused by the nanoparticles can synergize each other with reducing the side effects of PZQ.

### DNA damage by single cell electrophoresis assay

**Figures 9 and 10:**
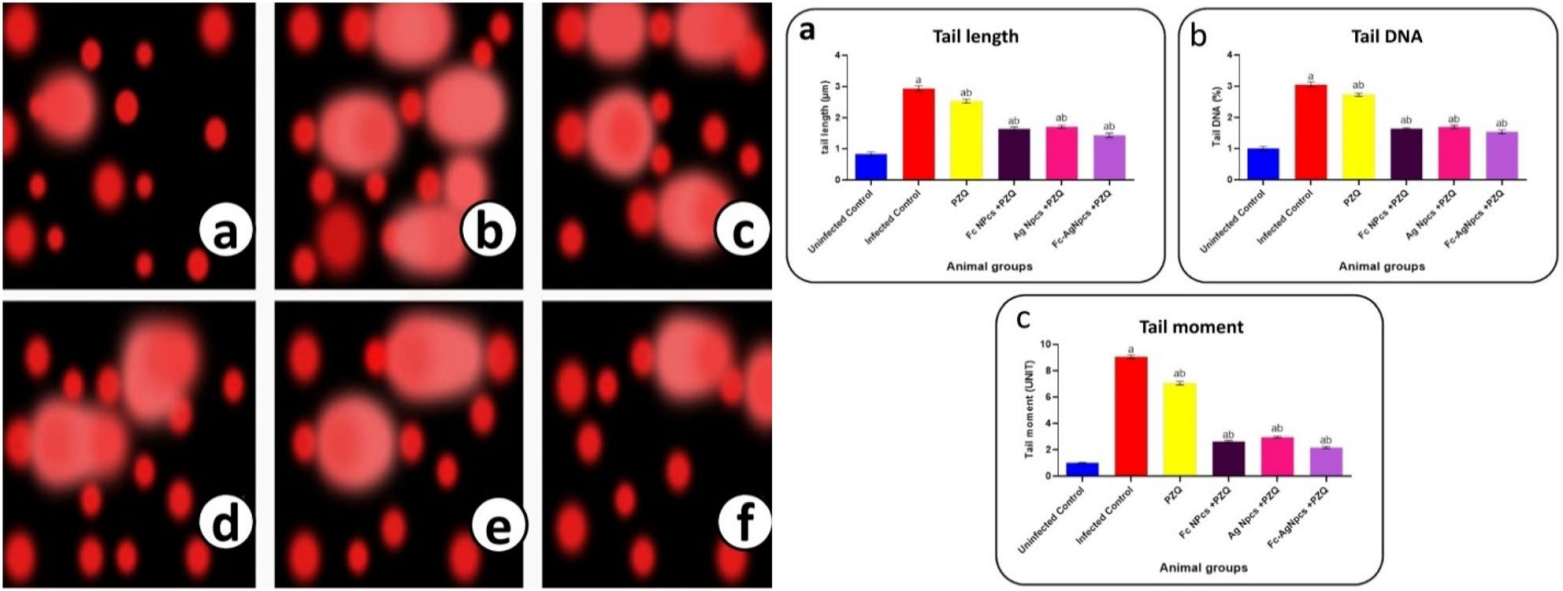
DNA damage as; Fig. 9 (**a**): Uninfected group showed normal damage, (**b**): Infected group showed abnormal damage, (**c**): PZQ showed abnormal damage, (**d**): Fc-NPCs + PZQ showed amelioration with damaged spots, (**e**): Ag-NPCs + PZQ showed amelioration in addition to damaged spots & (**f**): Fc-Ag NPCs + PZQ showed the best recovery status, (Scale bar = 100 µm). These results were also observed in Fig. 10 (**a, b & c**) in Tail length, Tail DNA & Tail moment in PZQ (Praziquantel), Fc-NPCs (*Ficus carica* nanoparticles), Ag-NPCs (Silver nanoparticles) & Fc-Ag NPCs (*Ficus carica* nanoparticles loaded on silver nanoparticles).

Figure 9a–f and Fig. 10(a–c) showed DNA damage of a single cell of hepatic tissue to record Tail length (TL), Tail DNA (DNA %) & Tail moment (TM) in infected mice and other tested groups.

#### The effect of different nanoparticle groups on DNA damage

The data showed normal spots in the uninfected group and abnormal spots in the infected group. The presented data showed amelioration in Ag-NPCs, Fc-NPCs and Fc-Ag NPCs in reducing DNA damage spots (Tail length, Tail moment and DNA %) compared with the infected group.

#### Praziquantel treatment effects on DNA damage

The PZQ stimulated cell apoptosis, which in turn caused DNA damage showed cell death as abnormal spots and appeared in the TL, TM, and DNA%.

#### The effect of *Ficus carica* nanoparticles on DNA damage

The current study observed that the active phytochemicals of *Ficus carica* leaves had a protective role against reactive oxygen species (ROS) and reduced DNA damage.

#### The effect of silver nanoparticles on DNA damage

Reference^42^ discussed that Ag-NPCs had a protective effect on DNA against Triclosan-induced DNA damage.

#### The effect of *Ficus carica* nanoparticles loaded on silver nanoparticles on DNA damage

The combination of phytochemical nanoparticles *F. carica* and silver nanoparticles showed better results than each of them separately, and this showed decay destruction in DNA so, it might be able to antagonize PZQ side effects that appeared in the best protected-treated mice group. Also, prophylactic Ag-NPCs recorded a reduction in DNA damage by schistosomal cercariae.

## Conclusion

**Figure 11:**
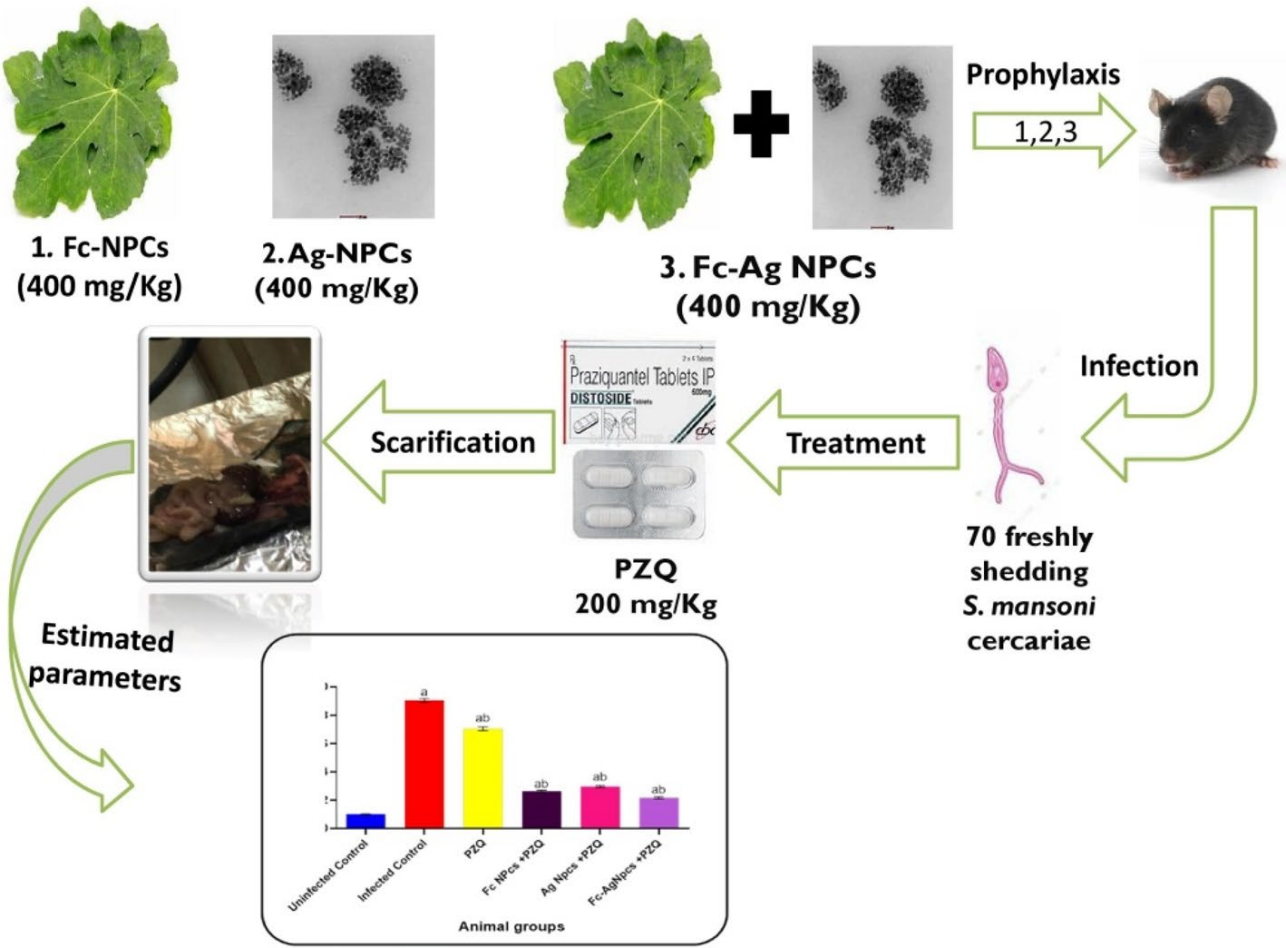
The appearance of amelioration by nanoparticles’ components in obtained results after *Schistosoma mansoni* infection.

The current study proved the protective effect of active phytochemicals specifically phenolic compounds of *Ficus carica* leaves nanoparticles such as flavonoids and phenolic acids; these compounds were known for their antioxidant properties, which helped in neutralizing harmful free radicals in the body, thereby protecting cells from oxidative stress and reducing the risk of chronic diseases against bilharzia infection. This showed its effect on improving liver function indicators, anti-oxidants, and anti-apoptotic additionally minimizing oxidative stress, pro-inflammatory, apoptotic markers also DNA damage. Undoubtedly, reduces the side effects of the PZQ drug. And don’t underestimate the effective role of silver nanoparticles singly and combined with the Fc-NPCs which may be able to increase the antagonistic effect for PZQ side effects that appeared in the combined group (Fc-Ag NPCs + PZQ); the current experiment was summarized in Fig. 11.

### Supplementary Information

Supplementary Tables.

## Data Availability

The datasets generated and analyzed during the current study are available in the file; its name is Supplementary file.
