# Supplementary material for: The synergistic effect of Ficus carica nanoparticles and Praziquantel on mice infected by Schistosoma mansoni cercariae
Source: Sci Rep. 2024 Aug 15;14:18944. doi: 10.1038/s41598-024-68957-9 (PMC11327331; doi:10.1038/s41598-024-68957-9)
Supplement: Supplementary file 1 — Supplementary Tables. [file 41598_2024_68957_MOESM1_ESM.docx]

**Table (1): It concerning to Figure 5 (Liver function tests)**

a: Significant as compared uninfected control group. b: Significant as compared with infected control group. Mean is considered significant when *p* ≤ 0.05.

| Animal groups  Parameters | Uninfected  control | Infected  control | PZQ | Fc-NPCs + PZQ | Ag-NPCs  + PZQ | Fc-Ag NPCs + PZQ |
| --- | --- | --- | --- | --- | --- | --- |
| ALT (U/L) | 28.33 ±1.76 | 198^a^ ±2.646 | 132^ab^ ±1.732 | 84^ab^ ±4.62 | 90.33^ab^ ±5.207 | 75.67^ab^ ±6.741 |
| AST (U/L) | 35  ±2.65 | 250^a^ ±17.3 | 155^ab^ ±24.9 | 112^ab^ ±10.4 | 115^ab^ ±13.2 | 98.3^b^ ±10.5 |
| ALP (U/L) | 54.67 ±6.39 | 208.3^a^ ±10.93 | 120^ab^ ±4.933 | 84.33^b^ ±5.239 | 86.33^b^ ±7.535 | 81^b^ ±5.859 |
| GGT (U/L) | 18 ±1.155 | 71.33^a^ ±4.91 | 44.33^ab^ ±1.856 | 28.33^b^ ±1.453 | 31.33^ab^ ±1.202 | 25.67^b^ ±2.028 |
| Alb (g/dl) | 4.2 ±0.231 | 1.243^a^ ±0.0977 | 2.24^ab^ ±0.095 | 3.13^ab^ ±0.091 | 3.073^ab^ ±0.089 | 3.433^ab^ ±0.088 |
| Bilirubin (mg/dl) | 0.28 ±0.095 | 1.253^a^ ±0.0742 | 0.84^ab^ ±0.051 | 0.467^b^ ±0.047 | 0.47^b^ ±0.050 | 0.44^b^ ±0.066 |

**Table (2): It concerning to Figure 6 (Oxidative stress markers & Anti-oxidants)**

a: Significant as compared uninfected control group. b: Significant as compared with infected control group. Mean is considered significant when *p* ≤ 0.05.

| Animal groups  Parameters | Uninfected  control | Infected control | PZQ | Fc-NPCs + PZQ | Ag-NPCs + PZQ | Fc-Ag NPCs + PZQ |
| --- | --- | --- | --- | --- | --- | --- |
| MDA (nmol/g) | 763.3 ±31.8 | 1120^a^ ±7.753 | 967.3^ab^ ±30.48 | 887^ab^ ±7.35 | 891.8^ab^ ±8.286 | 832.5^b^ ±31.79 |
| GSH  (nmol/g) | 4.42 ±0.297 | 1.04^a^ ±0.2117 | 2.655^ab^ ±0.1257 | 3.745^b^ ±0.279 | 3.6^b^ ±0.3147 | 3.998^b^ ±0.277 |
| SOD (U/g) | 184 ±6.92 | 106.7^a^ ±5.51 | 138.6^ab^ ±1.924 | 164.2^b^ ±5.96 | 158.1^b^ ±6.569 | 167.9^b^ ±9.947 |
| CAT (U/g) | 192.1 ±5.77 | 114^a^ ±7.08 | 138.5^ab^ ±2.149 | 162.1^ab^ ±3.67 | 159.6^ab^ ±4.738 | 172.9^b^ ±3.683 |

**Table (3): It concerning to Figure 7 (Pro-inflammatory markers)**

a: Significant as compared uninfected control group. b: Significant as compared with infected control group. Mean is considered significant when *p* ≤ 0.05.

| Animal groups  Parameters | Uninfected  control | Infected control | PZQ | Fc-NPCs + PZQ | Ag-NPCs + PZQ | Fc-Ag NPCs + PZQ |
| --- | --- | --- | --- | --- | --- | --- |
| CRP  (ng/ml) | 27.93 ±5.27 | 122.8^a^ ±6.227 | 95.67^ab^ ±3.528 | 57.73^ab^ ±7.147 | 65.83^ab^ ±3.977 | 46.67^b^ ±6.21 |
| IL-6 (pg/mg) | 2.543  ±0.09 | 7.37^a^ ±0.288 | 4.717^ab^ ±0.185 | 3.40^b^ ±0.283 | 3.54^b^ ±0.316 | 3.217^b^ ±0.21 |
| VCAM-1 (ng/mg) | 22.57  ±4.59 | 111.6^a^ ±7.085 | 81.57^ab^ ±5.235 | 52.77^ab^ ±3.683 | 53.3^ab^ ±6.022 | 43.8^b^ ±5.86 |
| ICAM-1 (pg/mg) | 111.9  ±6.84 | 293.2^a^ ±3.717 | 261.6^ab^ ±5.27 | 225.1^ab^ ±6.785 | 228.4^ab^ ±6.63 | 192.7^ab^ ±6.36 |

**Table (4): It concerning to Figure 8 (Pro-Apoptotic & Anti-apoptotic markers)**

a: Significant as compared uninfected control group. b: Significant as compared with infected control group. Mean is considered significant when *p* ≤ 0.05.

| Animal groups  Parameters | Uninfected control | Infected control | PZQ | Fc-NPCs + PZQ | Ag-NPCs + PZQ | Fc-Ag NPCs + PZQ |
| --- | --- | --- | --- | --- | --- | --- |
| P53 (pg/mg) | 145.7 ±6.51 | 272.5^a^ ±5.881 | 233^ab^ ±5.918 | 185.2^ab^ ±9.545 | 191.4^ab^ ±7.589 | 176^b^ ±7.81 |
| Bax (ng/mg) | 237.4 ±7.47 | 386.1^a^ ±6.264 | 330.9^ab^  ±11.17 | 253.2^b^ ±6.863 | 258.4^b^ ±6.15 | 250.5^b^ ±.005 |
| Bcl-2 (ng/mg) | 0.673 ±0.04 | 0.12^a^ ±0.01 | 0.320^ab^ ±0.021 | 0.533^b^ ±0.052 | 0.523^b^ ±0.038 | 0.667^b^ ±0.026 |
| Cytochrome-C (pg/mg) | 148.6 ±6.28 | 286.3^a^ ±5.774 | 242.8^ab^ ±6.406 | 199.6^ab^ ±6.622 | 220.2^ab^ ±5.959 | 176.2^b^ ±5.197 |
| Caspase-9 (pg/mg) | 378.3 ±33.34 | 1089^a^ ±37.16 | 863.3^ab^ ±15.62 | 607.3^ab^ ±35.83 | 617.7^ab^ ±23 | 476.1^b^ ±33.76 |
| Caspase-3 (ng/mg) | 1.70 ±0.12 | 8.07^a^ ±0.46 | 6.33^ab^ ±0.19 | 3.28^ab^ ±0.217 | 3.42^ab^ ±0.254 | 2.37^b^ ±0.228 |

**Table (5): It concerning to Figure 10 (Single cell gel electrophoresis assay parameters)**

a: Significant as compared uninfected control group. b: Significant as compared with infected control group. Mean is considered significant when *p* ≤ 0.05.

| Animal groups  Parameters | Uninfected  control | Infected control | PZQ | Fc-NPCs + PZQ | Ag-NPCs + PZQ | Fc-Ag NPCs + PZQ |
| --- | --- | --- | --- | --- | --- | --- |
| DNA tail length (µm) | 0.84  ±0.060 | 2.94^a^  ±0.085 | 2.537^ab^ ±0.061 | 1.647^ab^ ±0.054 | 1.713^ab^ ±0.046 | 1.433^ab^ ±0.067 |
| Tail DNA (%) | 1.01  ±0.061 | 3.057^a^  ±0.083 | 2.73^ab^ ±0.053 | 1.647^ab^ ±0.020 | 1.7^ab^ ±0.049 | 1.543^ab^ ±0.060 |
| Tail moment  (µm) | 1.017  ±0.043 | 9.05^a^  ±0.132 | 7.053^ab^ ±0.135 | 2.63^ab^ ±0.078 | 2.957^ab^ ±0.062 | 2.17^ab^ ±0.076 |
